# Membrane Signaling Induced by High Doses of Ionizing Radiation in the Endothelial Compartment. Relevance in Radiation Toxicity

**DOI:** 10.3390/ijms141122678

**Published:** 2013-11-18

**Authors:** Isabelle Corre, Maëva Guillonneau, François Paris

**Affiliations:** CRCNA-UMR Inserm U892-CNRS 6299-Institut de Recherche en Santé de l’Université de Nantes, Nantes 44007, France; E-Mails: maeva.guillonneau@etu.univ-nantes.fr (M.G.); fparis@nantes.inserm.fr (F.P.)

**Keywords:** radiotherapy, radiation toxicity, endothelium, plasma membrane, acid sphingomyelinase, ceramide

## Abstract

Tumor areas can now be very precisely delimited thanks to technical progress in imaging and ballistics. This has also led to the development of novel radiotherapy protocols, delivering higher doses of ionizing radiation directly to cancer cells. Despite this, radiation toxicity in healthy tissue remains a major issue, particularly with dose-escalation in these new protocols. Acute and late tissue damage following irradiation have both been linked to the endothelium irrigating normal tissues. The molecular mechanisms involved in the endothelial response to high doses of radiation are associated with signaling from the plasma membrane, mainly via the acid sphingomyelinase/ceramide pathway. This review describes this signaling pathway and discusses the relevance of targeting endothelial signaling to protect healthy tissues from the deleterious effects of high doses of radiation.

## 1. Introduction

In anticancer therapy, radiation therapy is a mainstay. It is largely used for the local control of tumor progression and to preserve organs. In radiation therapy, high-energy ionizing radiation is used to eradicate tumor cells. However, healthy cells located in the area neighboring the tumor also inevitably receive a considerable dose. This can cause damage to healthy tissues, which may appear immediately or later, if the patient survives. The number of cancer survivors is steadily increasing, thus it is important to assess how early and late radiation toxicity to normal tissues affects quality of life for these patients [1]. Because of this, the dose of radiation is restricted to avoid excess damage to healthy tissue. This has limited attempts to increase the therapeutic ratio (*i.e.*, maximization of tumor sterilization while sparing the surrounding normal tissue) as part of modern treatment.

Radiation delivery has significantly improved in recent decades, mainly through advances in computer technology. Improved tumor imaging combined with precise modulation of radiation beams has led to the development of intensity-modulated radiotherapy (IMRT), image-guided radiotherapy (IGRT) and high-dose hypofractionated stereotactic radiotherapy [2]. Among these new protocols, stereotactic body radiotherapy (SBRT) has emerged as a very promising tool, allowing delivery of limited fractions (1–4) of high doses of radiation (7.7 to 45 Gray (Gy)). This is possible because the tumor is very accurately localized. Recent clinical trials of SBRT revealed that it is feasible, effective and safe for the treatment of early primary tumors and oligometastatic diseases [3,4]. Although SBRT is designed to minimize irradiation of the healthy tissue surrounding the tumor, we currently have little feedback on early and late SBRT toxicity. This is mainly due to limited clinical data including long-term follow-up and to poor knowledge of the radiobiology of high doses of radiation [5]. The model conventionally used to predict tumor response and normal tissue toxicity from fractionated radiation, is the cell survival linear-quadratic model (LQ). This model appears to be too simplistic for SBRT, leading to overestimation of cell toxicity [6]. Indeed, the LQ model is essentially based on the capacity of normal cells to repair radiation-induced DNA damage. However, other molecular mechanisms may be involved and non-dividing cells present in normal tissues could also play a role [4].

**Figure 1:**
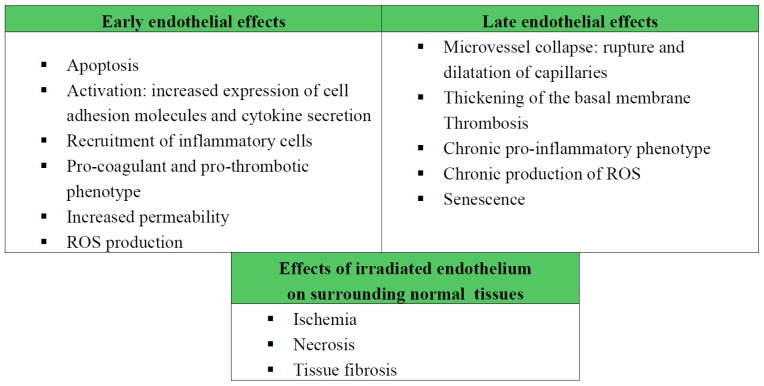
Early and late effects of ionizing radiations on endothelial cells. Relevance in tissue radiation toxicity.

Among this population of non-dividing cells, the microvascular endothelial compartment has clearly been shown to play a central role in radiation toxicity in healthy tissues. Several preclinical studies have established that radiation levels above 10 Gy cause endothelial cells to enter apoptotic cell death, resulting then in toxicity to the central nervous system [7] and to the small intestines [8]. Vascular injury has long been known to be the most common effect of exposing normal tissues to radiation, and the microvascular network is the most sensitive constituent of the vascular system [9]. The response of the microvasculature to radiation can be classed according to acute and late effects, both of which contribute to the initiation, progression and maintenance of tissue damage [10]. Following exposure to high doses of radiation (>10 Gy), a rapid wave of apoptosis appears as a hallmark of acute endothelial reaction. Surviving cells develop a dysfunctional vascular phenotype marked by excess secretion of pro-inflammatory cytokines, increased recruitment of blood cells and platelets, activation of the coagulation system and increased vascular permeability [11]. Late effects include microvessel collapse, thickening of the basement membrane, and persistence of an activated, pro-coagulant endothelial phenotype, which is ultimately senescent [12]. Through these effects, the microenvironment of normal tissue surrounding the irradiated tumor becomes a hypoxic, pro-inflammatory environment. This can cause damage to normal cells by inducing ischemia, necrosis and fibrosis (Figure 1).

Thus, even if the radiobiology of high doses of radiation is still incompletely understood, the endothelial compartment appears to be a major target, which when damaged can lead to radiation toxicity in normal tissues [13]. A single dose of 15–20 Gy induces molecular mechanisms of endothelial apoptosis, which requires activation of the acid sphingomyelinase (ASMase)/ceramide pathway [8,14]. This pathway is not triggered in tissues exposed to conventional fractionated radiotherapy [15]. Thus, these pioneering results demonstrated that an alternative radiation-induced signaling pathway exists, involving plasma membrane-initiated but nuclear-independent events [16]. The initial evidence for membrane signaling initially originated from studies on cancer cells exposed to low doses of radiation, where tyrosine kinase receptors epithelial growth factor-receptor (EGF-R), phosphoinositide 3-kinase PI3K/AKT and the Ras/Raf/mitogen activated protein kinase (MAPK) pathway were shown to mediate radiation sensitivity [17]. The role of membrane signaling in the response to radiation has now been clearly demonstrated, and the ASMase/ceramide signaling triggered in endothelium exposed to single high doses of radiation has been shown to play a major role.

This review will discuss what is currently known about the molecular signaling pathways induced in the endothelial compartment by high doses of radiation, such as those delivered by SBRT. Emphasis is placed on plasma membrane signaling events. Potential strategies to preserve and protect the microvasculature, thus preventing radiation toxicity in normal tissues, are also discussed.

## 2. Radiation-Induced Plasma Membrane Signaling in the Endothelial Compartment

### 2.1. Activation of the Acid Sphingomyelinase Pathway

#### 2.1.1. Translocation and Activation of ASMase

Cellular studies were the first to demonstrated a rapid activation of the enzyme acid sphingomyelinase (ASMase) at the plasma membrane in bovine endothelial cells exposed to a high single dose of ionizing radiation [16]. Human lymphoblast deficient for ASMase enzyme [18] and endothelial cells from mice invalidated for the ASMase gene *smpd1* [7] were found to be resistant to radiation-induced apoptosis. Thus, a key role of the ASMase pathway in radiobiology signaling in the endothelium was established.

ASMase catalyses the hydrolysis of the sphingolipid sphingomyelin (SM) at the outer layer of the plasma membrane, producing ceramide [19]. *In vitro* studies initially showed that ASMase performs optimally at an acidic pH; this is consistent with the lysosomal localization of this protein. Nevertheless, ASMase is also active at the cell’s surface membrane, where the lipid composition of the membrane can alter the Km of the enzyme, allowing its activation at a higher pH [20]. Very recent data in endothelial cells demonstrate that an acidic microenvironment is created at the plasma membrane upon fusion with lysosomes, enabling ASMase activity [21]. The initial step in ASMase activation appears to be translocation from the lysosomes to the extracellular leaflet of the plasma membrane, where its substrate sphingomyelin is located. A broad range of stresses (cytokines, viruses, ultraviolet (UV)) has been shown to induce ASMase trafficking in different cell types [22–24]. The rapid translocation of ASMase to the cell surface in endothelial cells exposed to 15 Gy appears to be a pre-requisite triggering intracellular apoptotic signals in these cells [25]. Several studies have investigated the molecular pathways involved in ASMase translocation from secretory lysosomes to the external face of the plasma membrane. These studies showed that various proteins of the exocytotic machinery, in particular the t-SNARE protein syntaxin-4 [26] and the protein dysferlin in endothelial cells [27], participate in lysosome fusion with membranes, therefore leading to ASMase translocation.

Despite intensive research into ASMase signaling, the molecular mechanisms causing activation of this enzyme are still not fully elucidated. ASMase might be activated in several different ways, depending on cells and stimuli. For example, proteases of the caspases family have been shown to regulate ASMase activation. In pro-apoptotic Fas/Fas ligand signaling, caspase-8 promotes ASMase translocation and activity [24], whereas upon tumor necrosis factor-α (TNF-α) signaling, caspase-7 can activate ASMase activation by direct interaction and proteolytic cleavage [28]. ASMase has also been found to be regulated by phosphorylation, with recombinant PKC-δ phosphorylating ASMase on serine-508. This phosphorylation appears to be necessary for the protein translocation and activation in carcinoma cells [29]. In stress conditions, ASMase could be regulated by redox mechanisms, and several studies support this hypothesis: oxidation at cysteine-629 in the *C*-terminal portion of ASMase leads to dimerization and increases its enzymatic activity [30], while scavengers for reactive oxygen species (ROS) prevent activation of ASMase by death receptors [23], UV-C [22] or Cu^2+^[31]. Reactive nitrogen species (RNS) can also specifically activate an acidic sphingomyelinase in airway epithelium [32]. In endothelial cells, the contribution of ROS and redox signaling to ASMase activation has been proven by Li and co-workers [33], who described ROS-dependant ASMase activation, as well as a feed-forward loop amplifying ROS production, which is detailed below. Ionizing radiation can be considered as a receptor-independent stimulus activating ASMase signaling. The first intracellular radiochemical event induced by ionizing radiation consists of a rapid burst of ROS, as a result of H_2_O radiolysis [34]. Thus, it is possible that activation of ASMase upon exposure to radiation might be provoked by oxidation through ROS produced by radiolysis. Indeed, anti-oxidants such as glutathione and *N-*acetylcysteine (NAC) impair plasma membrane translocation and activation of ASMase in microvascular endothelial cells exposed to 15 Gy [35].

#### 2.1.2. Ceramide, a Plasma Membrane Reorganizer

In response to radiation, ASMase activation results in the rapid and transient production of the sphingolipid ceramide. This bioactive lipid is an evolutionarily conserved second messenger, composed of a fatty acyl chain of variable length (the most common mammalian ceramides contain 14 to 24 carbon atoms) bound to the amino group of a sphingosine [36]. Ceramides are among the least polar and most hydrophobic sphingolipids and possess unique biophysical properties. In artificial membranes, ceramide molecules tend to spontaneously self-associate, excluding other lipids [37,38]. This creates small membrane ceramide-enriched domains, which can spontaneously coalesce to generate larger domains [36].

Biological membranes typically contain sphingolipids, cholesterol and other glycophospholipids. In this context, sphingolipids interact with each other through their hydrophilic part and with cholesterol molecules through their ceramide moiety forming hydrogen bonds and Van der Waals interactions with the sterol ring. These tight interactions promote the lateral association of sphingolipids and cholesterol molecules and result in the appearance of sphingolipid- and cholesterol-enriched domains, also known as membrane lipid rafts (LRs) in the cellular membrane [39]. Based on this model, and with regard to the biophysical properties of ceramide, an increase in membrane ceramide content would provoke extensive spatial reorganization, resulting from the fusion of membrane LRs into large ceramide-enriched domains. Indeed, several studies have shown these large LRs to occur in cells in response to different stimuli (CD95, CD40-L, viruses, chemotherapeutics, UV, *etc*. [40]). All these stimuli are known to trigger ceramide generation by ASMase. Biochemical fractionation and confocal fluorescent microscopy experiments have clearly demonstrated that LRs participate in signal transduction, as they serve to cluster signaling receptors and molecules in the plasma membrane, facilitating their activation [41]. In the context of radiation, these membrane LRs were first shown to be linked to the radiation sensitivity of human carcinoma cells [42]. This underlines the importance of the plasma membrane organization in ceramide-enriched LRs in the cellular response to ionizing radiation. In the endothelial compartment, the ASMase/ceramide pathway was rapidly identified as essential to initiating radiation-induced apoptosis [16]. This was recently proven, once again showing how membrane reorganization into ceramide-enriched large domains contributes to driving transmission of radiation-induced signaling [43].

The first role identified for ceramide-enriched domains was in the pro-apoptotic signaling pathway mediated by death receptors like CD95 and CD40 [44,45]. These domains trap and cluster receptors and intracellular signaling molecules, with consequent formation of an apoptotic “signalosome”, which can efficiently transmit and amplify the signal inside the cell. Aggregation and trapping of death receptors in ceramide-enriched domains limits their lateral diffusion, thus stabilizing receptor-ligands interaction [41]. In response to radiation in endothelial cells, ceramide-enriched LRs are also essential in transmitting apoptotic signals; this can be prevented by hindering the formation of these domains. Thus, both an anti-ceramide antibody [43] and LRs disruptors (filipin, methyl-β-cyclodextrin (MβCD)) [35] protect endothelial cells from radiation-induced cell apoptotic cell death. However, the intracellular signaling pathways involved have yet to be fully deciphered.

The best-characterized signaling molecules in ceramide-enriched domains are clusters of death receptors [44]. Very few other molecules have yet been specifically identified in these domains, except in endothelial cells, where subunits gp91^phox^ and p47^phox^ of the nicotinamide adenine dinucleotide phosphate (NADPH) oxidase (NOX) are concentrated in ceramide-enriched domains following ASMase activation [33,46]. Endothelial NOX is a multi-enzymatic complex involved in the reduction of molecular oxygen and generation of superoxide radicals, O_2_^•−^, is now considered as the primary enzymatic source of vascular ROS. The concept of redox lipid rafts was initially proposed to describe membrane LRs where NOX components were recruited, clustered and activated, therefore leading to ROS production [47]. The ASMase/ceramide pathway now appears essential to the formation of these redox domains [33], as ceramide-enriched domains promote aggregation and activation of the NOX complex. The initial functions attributed to structurally organized membrane domains LRs was the transmission and the amplification of intracellular signals [48]. In the case of redox LRs, a similar feed-forward amplification mechanism was proposed in endothelial cells [33], as ROS produced by NOX within ceramide-enriched domains may further enhance ASMase activation through an oxidative mechanism [30]. This would result in enhanced formation of ceramide LRs, thus amplifying the whole system. Activation of NOX through such domains is induced in the endothelial compartment in response to death factors Fas-L, TNF-related apoptosis-inducing ligand (TRAIL) and endostatin [33,49,50]. Ionizing radiation also activates NOX in microvascular endothelial cells by an as yet unknown mechanism [51]. The ASMase/ceramide pathway might be involved in ionizing radiation-induced NOX activation, through formation of redox LRs. Hence, oxidative stress signaling initiated by ionizing radiation could be enhanced through the positive feedback from ASMase. By inducing the formation of ceramide and redox LRs, ASMase contributes to ROS production by NOX, which in turn could retro-activate ASMase. This could also explain how just few primary ionization events caused by the direct effects of radiation on water molecules, could be sufficiently amplified to cause rapid and robust activation of cellular signaling pathways.

#### 2.1.3. Ceramide, a Second Messenger

In addition to its involvement in reorganizing the outer leaflet of the plasma membrane, ceramide was also shown to directly interact with proteins on the cytoplasmic side of the membrane. This is possible as ceramide molecules can slowly flip from the outer- to the inner-side of the lipid bilayer [52]. *In vitro* studies revealed several signaling proteins, which are direct effectors of ceramide. The ceramide-activated protein phosphatase (CAPP), namely PP2A and PP1 [53], are specifically activated by specific binding to ceramide [54]. These proteins regulate various signaling proteins: retinoblastoma gene product *Rb*, Bcl-2, AKT, c-Jun, PKC-α. Ceramide has also been described as an activator of the kinase suppressor of Ras (KSR) protein [55], which phosphorylates the protein Raf-1 [56]. A specific ceramide-binding domain was identified in KSR. This domain seems to be required for translocation of the kinase to the membrane [57]. Another well-characterized direct target of ceramide is PKC-ζ which is directly bound and activated by natural long-acyl chain ceramides [58–60]. Activation of the aspartic protease cathepsin D by ceramide has been described [61]. This links ceramide to the mitochondrial apoptotic pathway via activation of the protein Bid. Recent studies also identified plasma membrane ions channels as novel targets of ceramide, in particular the storage-operated Ca^2+^ channel involved in controlling calcium influx [62,63] and voltage-gated Kv1.3 K^+^ channels [64] which modulate potassium efflux. To date, none of these pathways triggered by direct interaction between ceramide and intracellular molecules were initially described in endothelial cells. However, direct targets of ceramide including CAPP, KSR, PKC, and cathepsin D, are ubiquitously expressed and could potentially be regulated in the endothelial compartment. PP2A, in particular, is described as involved in disrupting endothelial barrier integrity [65], while cathepsin D plays a role in apoptosis and redox signaling [66] and PKC-ζ is involved in gene transcription and endothelial nitric oxide synthase (eNOS) activation [67].

### 2.2. The Rho Family of Small GTPases

In addition to its complex lateral organization as a lipid bilayer, the plasma membrane is also linked to the actin cytoskeleton through interaction with lipid or protein effectors, which tether actin filaments to its inner face. The cytoskeleton plays a major role in maintaining cell shape. Ionizing radiation has been clearly shown to alter cytoskeletal organization [68–70]. *In vitro* studies have highlighted different effects of radiation on the actin network, which vary depending on cell type and the dose delivered. In pulmonary microvascular endothelial cells, cytoskeletal disorganization with changes to actin filaments was observed following doses between 6 and 30 Gy [68,71]. In dermal microvascular cells, a dose of 15 to 20 Gy led to a rapid and persistent increase in stress fiber formation [69,70]. Actin structures are mainly reorganized through the action of specialized proteins, namely the Rho family of small GTPases, which includes RhoA, Rac and Cdc42 [72]. The role played by RhoA in the endothelial response to a single high dose of radiation (15–20 Gy) has been addressed by two separate studies [69,70]. Their results show a rapid and transient activation of RhoA. This activation then leads to reorganization of the actin cytoskeleton, marked by the formation of stress fibers, and resulting in increased endothelial permeability [69], enhanced formation of focal adhesions structures and altered migration [70]. Cdc42 GTPases has also been linked to activation of primary microvascular endothelial cells in response to ionizing radiation [73]. The mechanisms leading to GTPase activation still remain to be elucidated and no direct link between the ASMase/ceramide signaling pathway and Rho GTPases has been described in endothelial cells. Nevertheless, *in vitro* studies with exogenous ceramides (short- or natural long-chain forms) identified both RhoA [74] and Rac [75] as potential ceramide targets. All members of this GTPase family only become fully activated upon anchoring to the inner face of the plasma membrane through their hydrophobic isoprenoid (farnesyl or geranylgeranyl) moiety. Several studies have suggested that GTPases concentrate at the plasma membrane in LRs, where they become activated [76,77]. Therefore, Rho GTPases could be activated through signals originating in ceramide-enriched domains. Indeed, one study has shown Rac to be present in redox LRs in endothelial cells, where it forms part of the multi-enzymatic complex NOX [47].

## 3. Protecting Tissues from Radiation Toxicity by Targeting the Endothelium

**Figure 2:**
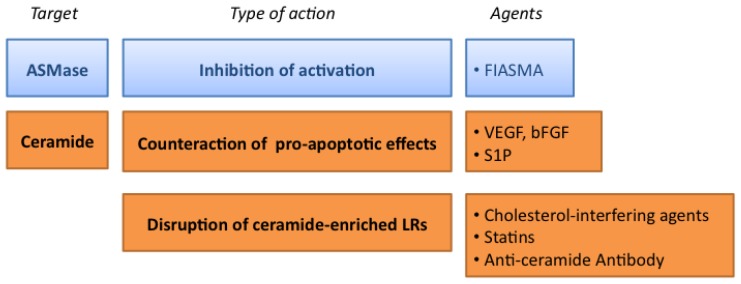
Targeting the ASMase pathway in the endothelium. See text for details.

The radiation doses above 10 Gy used in new hypofractionated radiotherapy strategies, such as SBRT, have a strong impact on the endothelium irrigating normal tissues. As mentioned before, early and late endothelial damage to the endothelium contribute to radiation toxicity in healthy tissues. Apoptotic loss of endothelial cells causes local ischemia, deregulated expression of adhesion molecules and secretion of cytokines. This creates a pro-inflammatory and a pro-coagulant environment. These acute effects often become persistent, leading to the establishment of a chronically dysfunctional endothelium, which can then lead to the formation of fibrotic tissue [78] and could even induce cardiovascular damage [79]. Therefore, protecting the endothelium from damage due to high-dose radiation is an attractive alternative therapeutic means to limit radiation toxicity in normal tissues. The relevance of the ASMase/ceramide pathway in the radiobiology of high-dose radiation prompts us to present a number of strategies to limit its activation (Figure 2).

### 3.1. Targeting the ASMase Activity

To limit the impact of signaling pathways dependent on the ASMase/ceramide pathway, an obvious approach would be to inhibit ASMase enzymatic activity itself. The three-dimensional structure of ASMase is not fully known and no crystallographic data are available yet. This has limited the development of direct specific inhibitors and those that exist show poor specificity. To date, only very few examples of direct ASMase inhibitors have been described, among which are phosphatidylinositol-3, 5-biphosphonate and natural xanthone compounds [80].

On the other hand, a broad group of molecules acting indirectly on ASMase activity has also been described. These are termed functional inhibitors of acid sphingomyelinase (FIASMA) [81]. They do not directly inhibit ASMase but act through an alternative mechanism based on the detachment of ASMase from the inner face of the lysosomal membrane, causing its proteolytic degradation. In normal conditions, positively charged protein ASMase attaches to the inner leaf of the lysosomal membrane through electrostatic interactions. FIASMA are lipophilic weak bases [81], thus they gain access inside the lysosome, where they become protonated and thus positively charged. This modifies the electrostatic properties of the inner surface of the lysosomal membrane and disrupts ASMase attachment, leading to its breakdown by lysosomal proteases. Seventy-two drugs approved by the Federal Drug Administration have been experimentally shown to inhibit ASMase activity, although none possess a unique specificity for ASMase [82]. Only one clinical study, involving cystic fibrosis treatment [83], used a drug from the FIASMA group, amitriptyline, specifically for its action on the ASMase.

### 3.2. Counteracting the Pro-Apoptotic Activity of Ceramide

The crucial role of endothelial cells in controlling radiation toxicity to healthy tissues has been demonstrated in many tissues, including the small intestines, brain, lung and parotid glands [7,8,18,84]. For example, C56BL/6 mice exposed to whole-body or whole-abdominal irradiation at 15 Gy died within days due to gastrointestinal syndrome (GIS). In these animals, microvascular endothelial apoptosis led to total collapse of the epithelial functionality [8]. Genetic invalidation of ASMase protects mice from GIS-related death following 15 Gy irradiation by preventing ceramide-induced apoptosis in the endothelial compartment. A similar protection from radiation-induced intestinal toxicity was achieved by intravenous injection of the pro-angiogenic factor bFGF. This molecule mainly targets the microvascular endothelium. This was the first evidence that tissues could be protected from radiation toxicity by targeting endothelial cells. However, this treatment seems compromised in human clinical trials, because it induces side effects including hypotension [85]. Recent data from the same group confirmed the potential role of pro-angiogenic molecules in limiting radiation toxicity in the endothelium in *in vitro* studies [25]. Treatment with vascular endothelial growth factor (VEGF) inhibited radiation-induced apoptosis through repression of the ASMase activation. The connection between the inhibition of ceramide-induced apoptosis and the late radiation toxicity remains unclear. However, ceramide is involved in long-term physiopathologies, such as cystic fibrosis [86]. Pharmalogical treatment with ASMase inhibitors or genetic heterozygosity of ASMase minimizes release of pulmonary inflammatory cytokines in mice developing cystic fibrosis. More studies must be done after exposure to ionizing radiation to properly establish the potential role of drugs targeting ceramide and preventing both acute and late toxicity.

The bioactive sphingolipid sphingosine-1-phosphate (S1P) is a ceramide metabolite, involved in numerous cellular functions, especially in cell survival. This pro-survival effect of S1P generally occurs when apoptotic signals are induced by ceramide [87–89]. Hence, the existence of a ceramide/S1P rheostat has been suggested to contribute to the cellular apoptosis/survival equilibrium [90]. In a murine intestinal radiation toxicity model, S1P was used as a ceramide antagonist, injected retro-orbitally into animals before whole-body irradiation at 15 Gy [91]. The radioprotective effects of S1P were clearly demonstrated, as S1P-injected were rescued from radiation-induced GIS. However, these animals died later from bone marrow aplasia. The specific cellular targets of S1P in the small intestine are microvascular endothelial cells and neither epithelial cell nor lymphocytes were protected from radiation-induced apoptosis in these animals. Further examination of the molecular radioprotective pathway regulated by S1P in endothelial cells revealed that it binds to receptors which in turn activate an AKT-dependent survival pathway [91]. Decoupling these seven-transmembrane-domain receptors using pertussis toxin or inhibiting the AKT-pathway with specific inhibitors prevents *in vitro* and *in vivo* S1P-induced radiation protection. However, S1P is a bioactive sphingolipid involved in a large number of cellular functions, including differentiation, cell trafficking, inflammation and migration. S1P also exhibits strong pro-angiogenic properties [92]. Because of this broad-ranging activity, S1P’s influence on late endothelial dysfunctions and related toxicity has yet to be determined.

In any case, it seems inconceivable to use pro-angiogenic drugs as radioprotectors of healthy tissues in a context of radiotherapy, as the primary effect of these molecules in an oncogenic environment would be to promote tumor angiogenesis, resulting in a burst in tumorigenesis and/or in metastasis [92]. Molecules targeting the endothelium but devoid of any pro-angiogenic activity would be suitable alternatives. This type of approach was explored by one study, where injection of an angiopoietin-1 variant, which exclusively targets the survival pathway in quiescent endothelial cells without affecting angiogenesis, was found to protect animals against radiation-induced endothelial apoptosis, leading to intestinal radioprotective effects [93]. S1P also targets only non-proliferating quiescent endothelial cells and not angiogenic tumoral endothelium [94], thus it could be a relevant avenue of investigation. Selective protection of quiescent endothelium may provide opportunities to define selective radioprotectors for normal tissues.

### 3.3. Targeting the Formation of Ceramide-Enriched LRs

As mentioned above, the ASMase-generated ceramide contributes to transmitting intracellular signals by rearranging the plasma membrane and promoting the formation of ceramide-enriched domains. Hence, interfering with the formation of these domains is an attractive option to efficiently block signal transduction induced by plasma membrane ceramide. Manipulating lipid raft domains without irreversibly destroying the cell structure has proved difficult, and targeting sphingolipids, the major constituents of membrane LRs, is all but impossible. Cholesterol, which is also abundantly present in these membrane domains, has been extensively targeted in attempts to disrupt LRs, using two different types of interfering agents. Cholesterol-depletors such as MβCD, specifically remove cholesterol from membrane domains, while cholesterol-sequestrators, like filipin and nystatin, bind this lipid in LRs, decreasing the fluidity of these domains. This blocks the clustering and aggregation of signaling molecules [95]. However, none of these molecules can be considered as potential therapeutic drugs, as they are toxic and/or have non-specific effects.

Among available drugs, statins are well-characterized as lipid-lowering drugs, inhibiting the mevalonate pathway of cholesterol synthesis. This decreases the level of circulating plasma cholesterol. Statin treatment also reduces the amount of cholesterol in LRs leading to their disruption [96]. In endothelial cells, chronic exposure to lovastatin or atorvastatin leads to disruption of membrane LRs and consequently to spatial disorganization of the oxidized-low density lipoprotein (ox-LDL) receptor (LOX-1). This protects the endothelium from the adverse effects of ox-LDL [96]. A very recent study reinforces this notion of a protective effect of statins through regulation of the dynamics of ceramide-enriched domains [97]. In arterial endothelial cells exposed to ox-LDL, pretreatment with statins protects cells from oxidative stress by preventing the formation of ceramide-enriched platforms. The resulting inhibition of NOX assembly and activation reduces ROS production, thereby limiting the deleterious effects of ox-LDL [97]. In the context of ionizing radiation, statins also protect endothelial cells from radiation-induced damage. *In vitro*, they exert persistent anti-inflammatory and anti-thrombotic effects on irradiated endothelial cells [98]. Preclinical studies of a rodent model of radiation toxicity in the skin, showed that pravastatin treatment abolished radiation-induced endothelial dysfunctions and had therapeutic effects on irradiated skin [99]. Neither of these studies addresses how statins achieve these effects, but it is possible they cause disorganization of membrane ceramide-enriched platforms in the membrane by reducing available membrane cholesterol. By interfering with the mevalonate pathway, statins also block the synthesis of isoprenoid intermediates, which are essential for trafficking and activation of small GTPases at the membrane [100]. Thus, statins can also be considered to be indirect inhibitors of small GTPases. In microvascular endothelial cells, the effects of statins on radiation-induced RhoA activity have not yet been tested [69,70]. Nevertheless, statins may play a role in preventing radiation-induced Rho GTPase-dependent effects. In a model of radiation-induced intestinal fibrosis involving Rho proteins [101], pravastatin limits damage, possibly via inactivation of the RhoA/ROCK pathway [102].

Specifically targeting membrane ceramide to prevent radiation toxicity was recently achieved through the development and characterization of a specific anti-ceramide antibody [43]. The monoclonal 2A2 antibody binds to membrane ceramide molecules, blocks their redistribution and therefore prevents the formation of ceramide-enriched domains in endothelial plasma membranes. Without these structures, transmission of radiation-induced apoptotic signals is impaired. 2A2 antibody applied to the C56BL/6 model of radiation-induced intestinal toxicity also protects animals from death by GIS. Results from this study emphasize that intestinal crypts are protected from radiation-induced lethality by inhibition of endothelial cell apoptosis, through reduced formation of ceramide-enriched domains. Thus, this monoclonal antibody can be considered as the prototype of a new class of anti-ceramide therapeutics, potentially useful in radioprotection of healthy tissues.

## 4. Conclusions

**Figure 3:**
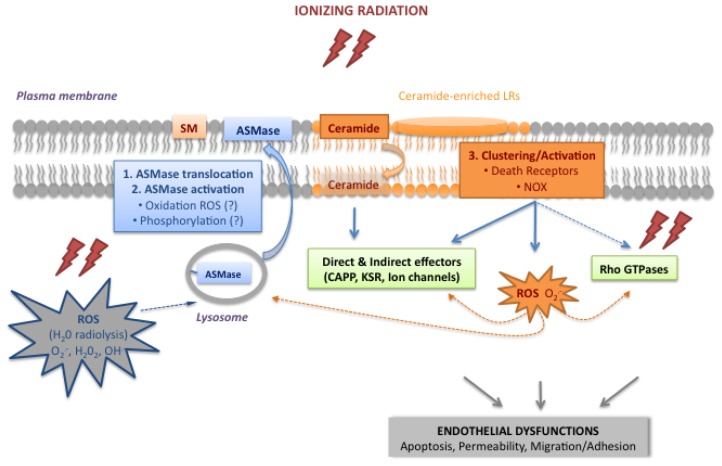
Diagram of the plasma membrane signaling pathways induced by ionizing radiation in endothelial cells. See text for details.

For decades, radiobiologists assumed that the DNA damage response was the only signaling pathway involved in the cellular response to ionizing radiation. While not disregarding this response, laboratory experiments and preclinical studies have now revealed additional biological processes involved in radiation toxicity in normal tissues exposed to a high single dose radiation. First, the crucial role of the microvascular microenvironment was established when rapid endothelial apoptosis and vascular collapse was shown to markedly enhance radiation-induced damage to normal cells. Second, the molecular mechanisms were characterized, highlighting the existence of a major signal transduction pathway, initiated at the plasma membrane but independent of nuclear events, requiring activation of the acid sphingomyelinase pathway. As illustrated in Figure 3, ASMase and ceramide are the main components of this radiation-induced endothelial signaling pathway. Membrane translocation of ASMase and its activation (Steps 1 and 2) produce ceramide. This transmits signals through lateral membrane reorganization, forming ceramide-enriched LRs domains (Step 3), or directly activates signaling molecules. An alternative endothelial signaling pathway involves the Rho family of small GTPases; these are suspected, but not proven, to be dependent on ceramide. In the endothelium, the intracellular ROS appear to play a key role in initiating, coordinating and amplifying plasma membrane events. The initial wave of ROS resulting from H_2_O radiolysis (ROS, in grey in Figure 3) might trigger the membrane ceramide pathway through ASMase activation. A second wave of ROS produced by ceramide-induced NOX activation (ROS, in orange in Figure 3) could then amplify the whole process and/or activate effectors. Altogether, this shows endothelial cells to mount a rather complex response to a high single dose of ionizing radiation, stressing the major role of the bioactive sphingolipid ceramide and the important contribution of ROS and the resulting oxidative stress.

Several strategies proposed to protect healthy tissue from radiation toxicity have been recently reviewed [103]: non-specific approaches using antioxidant molecules to neutralize radiation-induced oxidative stress, more specific treatments directly targeting normal tissue: *p53* modulation, injection of hormones or cytokine to stimulate proliferation, stem cell therapies, *etc*. Nevertheless, targeting the microvascular endothelial network is also a promising approach to limiting radiation toxicity to normal tissues. Inactivating signaling pathways involved in the endothelial response to radiation could be a suitable alternative, especially through therapies targeting the ASMase/ceramide pathway, which are currently in development.

## Abbreviations

ASMase: acid sphingomyelinase
bFGF: basic fibroblast growth factor
CAPP: ceramide-activated protein phosphatase
EGF-R: epithelial growth factor-receptor
eNOS: endothelial nitric oxide synthase
FIASMA: functional inhibitors of acid sphingomyelinase
GIS: gastrointestinal syndrome
Gy: Gray
IGRT: image-guided radiotherapy
IMRT: intensity-modulated radiotherapy
KSR: kinase suppressor of Ras
ox-LDL: oxidized-low density lipoprotein
LRs: lipid rafts
LOX-1: oxidized-low density lipoprotein receptor
MAPK: mitogen activated protein kinase
MβCD: methyl-β-cyclodextrin
NADPH: nicotinamide adenine dinucleotide phosphate
NAC: *N*-acetylcysteine
NOX: nicotinamide adenine dinucleotide phosphate oxidase
PI3K: phosphoinositide 3-kinase
ROS: reactive oxygen species
RNS: reactive nitrogen species
SBRT: stereotactic body radiotherapy
S1P: sphingosine-1-phosphate
SM: sphingomyelin
TNF-α: tumor necrosis factor-α
TRAIL: TNF-related apoptosis-inducing ligand
UV: ultraviolet
VEGF: vascular endothelial growth factor.
